# Investigation of Communication Type and Individual Characteristics : A Longitudinal HRS Analysis

**DOI:** 10.1093/geroni/igab046.3344

**Published:** 2021-12-17

**Authors:** Kaleena Odd, Sarah Hubner, Hyeon Jung Kim,, Julie Blaskewicz Boron

**Affiliations:** University of Nebraska Omaha, Omaha, Nebraska, United States

## Abstract

Older adults are at increased risk for loneliness/isolation, particularly with new COVID-19 recommendations; however, communication may help mitigate these negative perceptions. Reductions in loneliness/isolation may also significantly improve quality of life and well-being for vulnerable populations. Thus, the purpose of this study was to investigate the relationships between communication, individual characteristics, and time, to provide a clearer understanding of communication patterns in a longitudinal cohort. Participants (N=2351) with no missing data on any variables of interest (across time-points) were pulled from the Health and Retirement Study’s Consumption and Activity’s Mail Survey (waves collected: 2013, 2015, 2017). When last reported (2016/17), respondents were an average age of 70.14(SD=9.9), were generally female (63.0%) and white (75.7%). Analyses included longitudinal investigation, normality tests, and regression. Assumptions were violated in ANOVA; results of a Kruskal-Wallis test revealed that there were no significant changes in the distribution of in-person or distanced communication across the three waves. Individual responses were then averaged and standardized across waves (per participant for each outcome variable). In-person communication regression results reveal that being female positively predicted in-person conversation volume (B=0.23,p<.001) as did increasing number of years in school (B=0.03,p<.001), while being non-white negatively predicted in-person conversation (B=-0.301,p<.001). Distanced communication regression results reveal being female positively predicted volume of distanced communication (B=0.381,p<.001); however, being non-white and younger positively predicted increased volume of distanced communication (B=0.241,p<.001; B=0.005,p<.001, respectively). Given the varied communication patterns, future research should consider explanatory mechanisms in addition to investigating changes as a result of the ongoing pandemic.

